# Supplementary material for: The cardiovascular polypill as baseline treatment improves lipid profile and blood pressure regardless of body mass index in patients with cardiovascular disease. The Bacus study
Source: PLoS One. 2023 Aug 25;18(8):e0290544. doi: 10.1371/journal.pone.0290544 (PMC10456133; doi:10.1371/journal.pone.0290544)
Supplement: S1 Fig — ASA: acetylsalicylic acid; RAAS: renin-angiotensin-aldosterone system. (PDF) [file pone.0290544.s001.pdf]

S1 Fig. Overall proportion of patients receiving ASA, RAAS inhibitors and statins A) prior to initiating or switching to the CV polypill strategy, at baseline, and at the end of follow-up and B) by BMI group prior to CV polypill initiation.

A

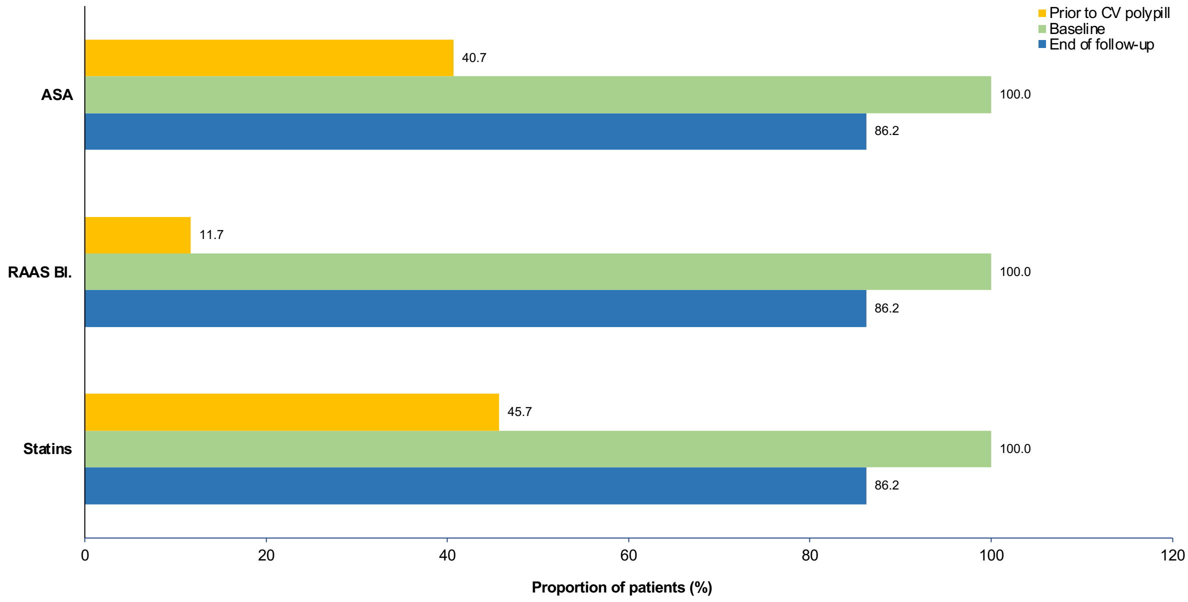

B

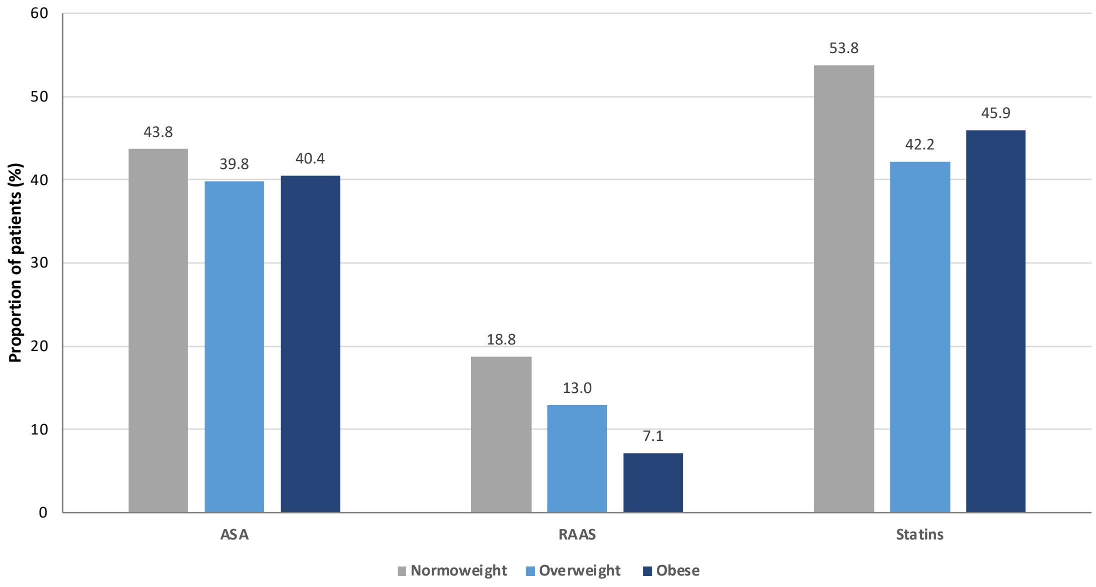

ASA: acetylsalicylic acid; RAAS: renin-angiotensin-aldosterone system
