# Supplementary material for: The cardiovascular polypill as baseline treatment improves lipid profile and blood pressure regardless of body mass index in patients with cardiovascular disease. The Bacus study
Source: PLoS One. 2023 Aug 25;18(8):e0290544. doi: 10.1371/journal.pone.0290544 (PMC10456133; doi:10.1371/journal.pone.0290544)

S2 Fig. Proportion of patients by BMI group receiving equipotent, more potent, and less potent statins (A) or RAAS inhibitor (B) prior to initiating or switching to the CV polypill strategy.

A

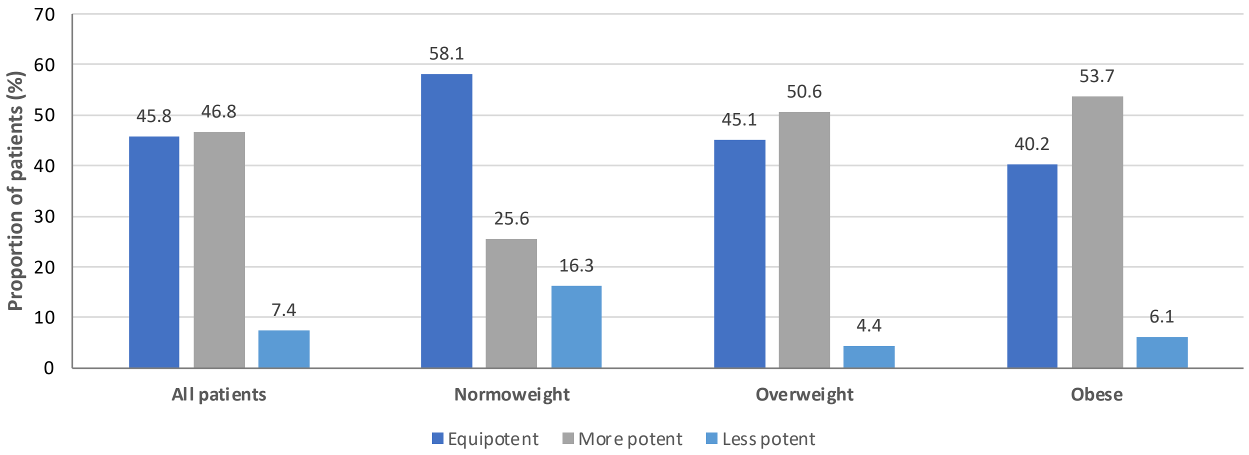

B

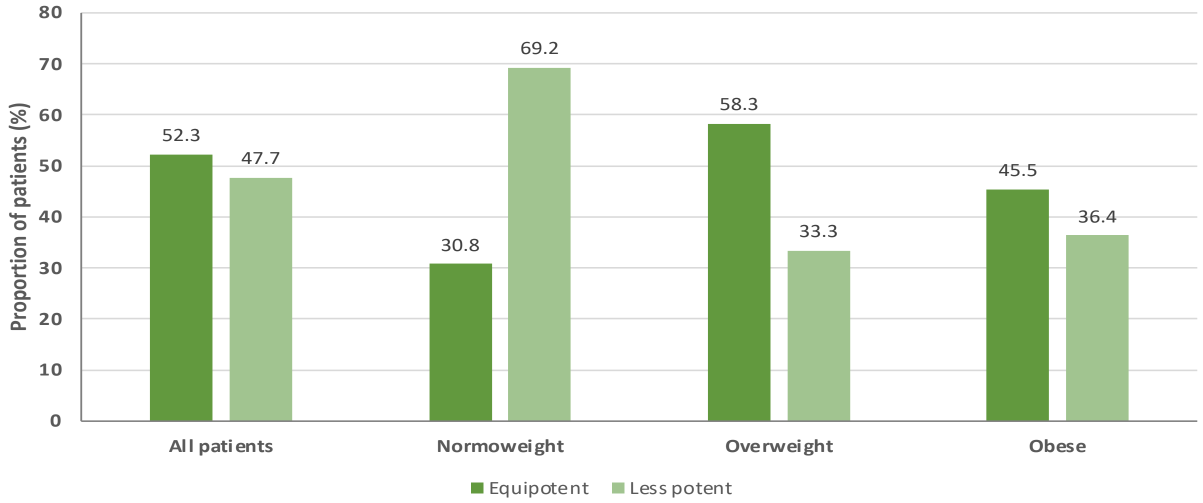

Supplement: S2 Fig — (PDF) [file pone.0290544.s002.pdf]
