# Supplementary material for: The cardiovascular polypill as baseline treatment improves lipid profile and blood pressure regardless of body mass index in patients with cardiovascular disease. The Bacus study
Source: PLoS One. 2023 Aug 25;18(8):e0290544. doi: 10.1371/journal.pone.0290544 (PMC10456133; doi:10.1371/journal.pone.0290544)
Supplement: S3 Fig — ASA: acetylsalicylic acid; BB: beta blockers; DIU: diuretics; CCB: calcium channel blockers. (PDF) [file pone.0290544.s003.pdf]

S3 Fig. Evolution of concomitant medications in the overall population during the different assessed periods.

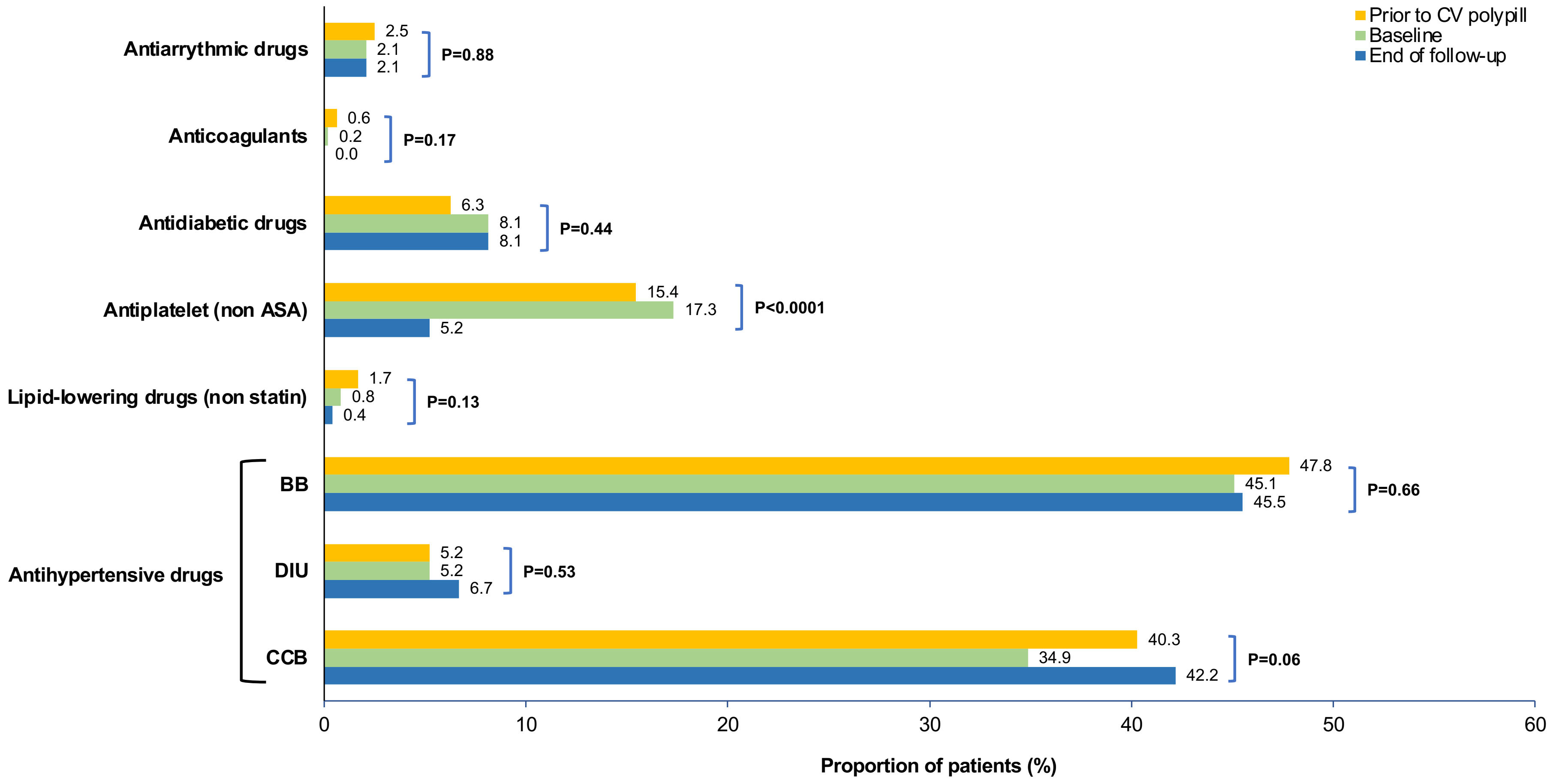

ASA: acetylsalicylic acid; BB: beta blockers; DIU: diuretics; CCB: calcium channel blockers
