# Supplementary material for: The cardiovascular polypill as baseline treatment improves lipid profile and blood pressure regardless of body mass index in patients with cardiovascular disease. The Bacus study
Source: PLoS One. 2023 Aug 25;18(8):e0290544. doi: 10.1371/journal.pone.0290544 (PMC10456133; doi:10.1371/journal.pone.0290544)
Supplement: S5 Fig — ***P<0.0001; **P<0.01; *P<0.05. BP: blood pressure; LDL-c: low-density lipoprotein cholesterol; w: with; w/o: without. (PDF) [file pone.0290544.s005.pdf]

S5 Fig. Achievement of target levels of LDL-c and recommended levels of triglycerides (A) and blood pressure (B) with the CV polypill compared to baseline in patients >65 years.

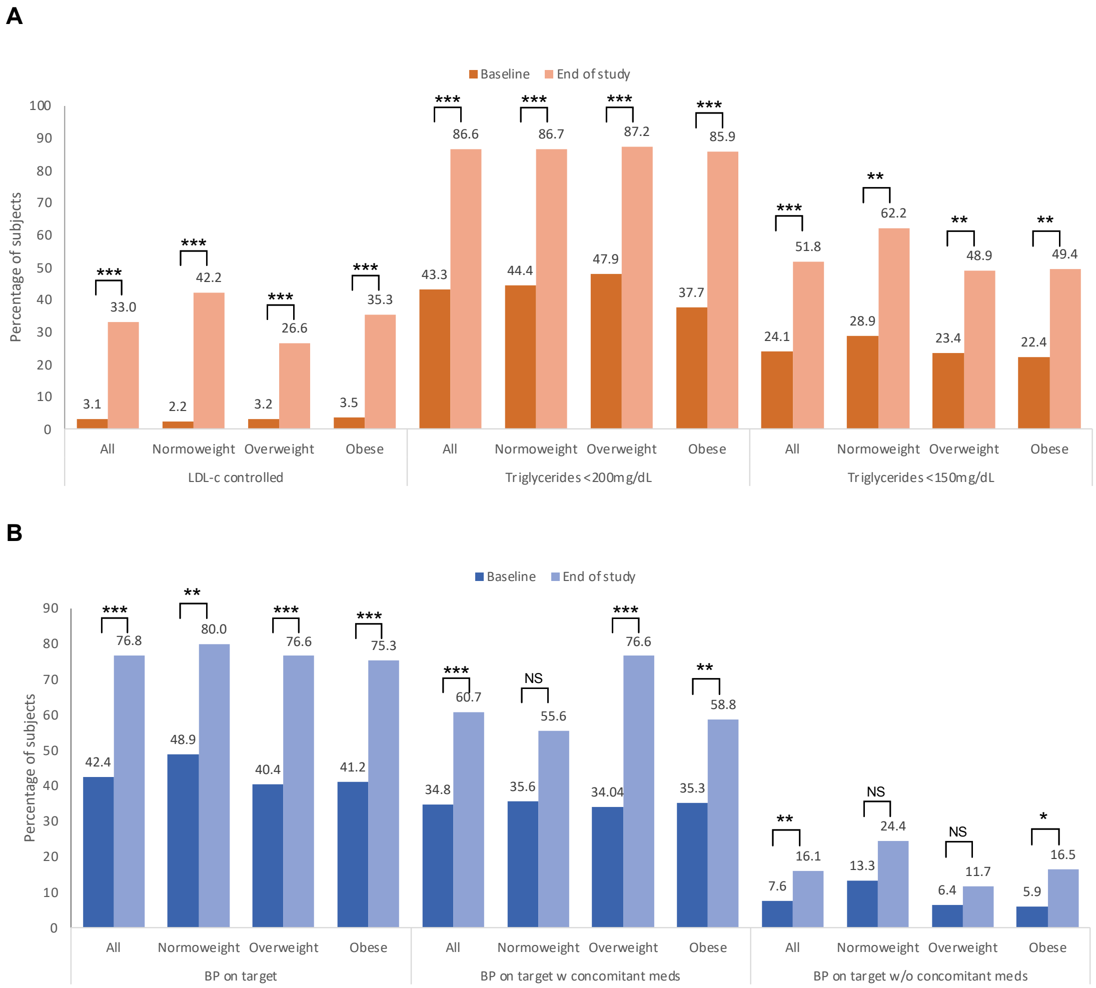

***P<0.0001; **P<0.01; *P<0.05

BP: blood pressure; LDL-c: low-density lipoprotein cholesterol; w: with; w/o: without
