# Supplementary material for: The cardiovascular polypill as baseline treatment improves lipid profile and blood pressure regardless of body mass index in patients with cardiovascular disease. The Bacus study
Source: PLoS One. 2023 Aug 25;18(8):e0290544. doi: 10.1371/journal.pone.0290544 (PMC10456133; doi:10.1371/journal.pone.0290544)
Supplement: S1 Table — (PDF) [file pone.0290544.s006.pdf]

S1 Table. Reasons for CV polypill discontinuation in the overall population and by BMI subgroup.

	All (N=66)	Normal weight (n=12)	Overweight (n=28)	Obese (n=26)	P value
**Cough**	21 (31.8%)	3 (25.0%)	9 (32.1%)	9 (34.6%)	0.886
**Hypotension**	4 (6.1%)	2 (16.6%)	0 (0%)	2 (7.8%)	0.096
**Economic reasons**	3 (4.5%)	0 (0%)	2 (7.1%)	1 (3.8%)	1.000
**Reason not reported**	3 (4.5%)	0 (0%)	2 (7.1%)	1 (3.8%)	1.000
**Atrial fibrillation because of cardiac arrhythmia**	2 (3.0%)	1 (16.6%)	0 (0%)	1 (3.8%)	0.176
**Severe heart failure**	2 (3.0%)	0 (0%)	2 (7.1%)	0 (0%)	0.660
**Bleeding**	2 (3.0%)	0 (0%)	1 (3.6%)	1 (3.8%)	1.000
**Pain**	2 (3.0%)	0 (0%)	1 (3.6%)	1 (3.8%)	1.000
**Could not find the medication**	2 (3.0%)	0 (0%)	1 (3.6%)	1 (3.8%)	1.000
**Atrial fibrillation**	1 (1.5%)	1 (8.3%)	0 (0%)	0 (0%)	0.182
**Heart failure**	1 (1.5%)	0 (0%)	1 (3.6%)	0 (0%)	1.000
**Acne and cough**	1 (1.5%)	0 (0%)	0 (0%)	1 (3.8%)	0.575
**Angina**	1 (1.5%)	0 (0%)	1 (3.6%)	0 (0%)	1.000
**Change to social security system**	1 (1.5%)	0 (0%)	1 (3.6%)	0 (0%)	1.000
**Headache**	1 (1.5%)	0 (0%)	1 (3.6%)	0 (0%)	1.000
**Bypass surgery**	1 (1.5%)	1 (8.3%)	0 (0%)	0 (0%)	0.182
**Convulsive crisis**	1 (1.5%)	0 (0%)	1 (3.6%)	0 (0%)	1.000

	All (N=66)	Normal weight (n=12)	Overweight (n=28)	Obese (n=26)	P value
**Extreme weakness**	1 (1.5%)	0 (0%)	0 (0%)	1 (3.8%)	0.575
**Dyspnea**	1 (1.5%)	0 (0%)	0 (0%)	1 (3.8%)	0.575
**Gastrointestinal disease**	1 (1.5%)	1 (8.3%)	0 (0%)	0 (0%)	0.182
**Tooth removal**	1 (1.5%)	0 (0%)	0 (0%)	1 (3.8%)	0.575
**Lack of efficacy**	1 (1.5%)	0 (0%)	0 (0%)	1 (3.8%)	0.575
**Gastritis**	1 (1.5%)	1 (8.3%)	0 (0%)	0 (0%)	0.182
**Hyperglycemia**	1 (1.5%)	0 (0%)	0 (0%)	1 (3.8%)	0.575
**Persistent Hypertension**	1 (1.5%)	0 (0%)	1 (3.6%)	0 (0%)	1.000
**Urinary incontinence/weight loss**	1 (1.5%)	0 (0%)	1 (3.6%)	0 (0%)	1.000
**Intolerance to aspirin**	1 (1.5%)	0 (0%)	1 (3.6%)	0 (0%)	1.000
**Surgery**	1 (1.5%)	0 (0%)	0 (0%)	1 (3.8%)	0.575
**Patient never returned to control visits**	1 (1.5%)	0 (0%)	0 (0%)	1 (3.8%)	0.575
**Cardiorespiratory arrest**	1 (1.5%)	0 (0%)	0 (0%)	1 (3.8%)	0.575
**Personal reasons**	1 (1.5%)	0 (0%)	1 (3.6%)	0 (0%)	1.000
**Throat dryness**	1 (1.5%)	1 (8.3%)	0 (0%)	0 (0%)	0.182
**Atrial fibrillation/cough**	1 (1.5%)	1 (8.3%)	0 (0%)	0 (0%)	0.182
**Ulcer**	1 (1.5%)	0 (0%)	1 (3.6%)	0 (0%)	1.000
