# Supplementary material for: The cardiovascular polypill as baseline treatment improves lipid profile and blood pressure regardless of body mass index in patients with cardiovascular disease. The Bacus study
Source: PLoS One. 2023 Aug 25;18(8):e0290544. doi: 10.1371/journal.pone.0290544 (PMC10456133; doi:10.1371/journal.pone.0290544)
Supplement: S2 Table — BMI: body mass index; DBP: diastolic blood pressure; HDL-c: high-density lipoprotein cholesterol; LDL-c; low-density lipoprotein cholesterol; SBP: systolic blood pressure; SD: standard deviation; TC: total cholesterol; TG: triglycerides. (PDF) [file pone.0290544.s007.pdf]

S2 Table. Evolution of concomitant medications during the different assessed periods by BMI group.

Drug class, n (%)		All (N=479)	Normal weight (n=80)	Overweight (n=216)	Obese (n=183)	P-value
**Antihypertensive drugs**						
Calcium channel blockers						
	Prior to switch	193 (40.29)	35 (43.75)	75 (34.72)	83 (45.35)	0.077
	Baseline	167 (34.86)	30 (37.5)	67 (31.01)	70 (38.25)	0.276
	End of follow-up	202 (42.17)	38 (47.5)	81 (37.5)	83 (45.35)	0.163
Diuretics						
	Prior to switch	25 (5.21)	5 (6.25)	10 (4.62)	10 (4.62)	0.841
	Baseline	25 (5.21)	7 (8.75)	9 (4.16)	9 (4.91)	0.272
	End of follow-up	32 (6.68)	6 (7.5)	16 (7.40)	10 (5.46)	0.704
Beta blockers						
	Prior to switch	229 (47.80)	37 (46.25)	112 (51.85)	80 (43.71)	0.257
	Baseline	216 (45.09)	36 (45)	106 (49.07)	74 (40.43)	0.225
	End of follow-up	218 (45.51)	32 (40)	110 (50.92)	76 (41.53)	0.095
**Lipid-lowering drugs**						
	Prior to switch	8 (1.67)	3 (3.75)	2 (0.92)	3 (1.63)	0.178
	Baseline	4 (0.83)	3 (3.75)	1 (0.46)	0 (0.0)	**0.016**
	End of follow-up	2 (0.41)	1 (1.25)	1 (0.46)	0 (0.0)	0.452
**Antiplatelet (non-ASA) agents**						
	Prior to switch	74 (15.44)	11 (13.75)	38 (17.59)	25 (13.66)	0.501
	Baseline	83 (17.32)	9 (11.25)	43 (19.90)	31 (16.93)	0.213
	End of follow-up	25 (5.21)	3 (3.75)	13 (6.02)	9 (4.9)	0.718
**Antidiabetic drugs**						
	Prior to switch	30 (6.26)	4 (5)	15 (6.94)	11 (6.01)	0.816
	Baseline	39 (8.14)	6 (7.5)	20 (9.25)	13 (7.10)	0.716
	End of follow-up	39 (8.14)	6 (7.5)	20 (9.25)	13 (7.10)	0.716
**Anticoagulants**						
	Prior to switch	3 (0.62)	0 (0.0)	2 (0.92)	1 (0.54)	1.00
	Baseline	1 (0.20)	0 (0.0)	0 (0.0)	1 (0.54)	0.549
	End of follow-up	0 (0.0)	0 (0.0)	0 (0.0)	0 (0.0)	NA
**Antiarrhythmic drugs**						
	Prior to switch	12 (2.50)	1 (1.25)	6 (2.77)	5 (2.73)	0.857
	Baseline	10 (2.08)	0 (0.0)	6 (2.77)	4 (2.18)	0.365
	End of follow-up	10 (2.08)	1 (1.25)	5 (2.31)	4 (2.18)	0.844
