# Supplementary material for: The cardiovascular polypill as baseline treatment improves lipid profile and blood pressure regardless of body mass index in patients with cardiovascular disease. The Bacus study
Source: PLoS One. 2023 Aug 25;18(8):e0290544. doi: 10.1371/journal.pone.0290544 (PMC10456133; doi:10.1371/journal.pone.0290544)
Supplement: S3 Table — BMI: body mass index; DBP: diastolic blood pressure; HDL-c: high-density lipoprotein cholesterol; LDL-c; low-density lipoprotein cholesterol; SBP: systolic blood pressure; SD: standard deviation; TC: total cholesterol; TG: triglycerides. (PDF) [file pone.0290544.s008.pdf]

S3 Table. Baseline demographic and clinical characteristics of patients >65 years.

Characteristic	All (N=224)	Normal weight (n=45)	Overweight (n=94)	Obese (n=85)	P-value
**Age (years), mean (SD)**	74.6 (6.9)	76.3 (7.3)	75.1 (7.3)	73.1 (5.9)	0.033
**Gender (male), n (%)**	129 (57.8)	21 (46.6)	64 (68.8)	44 (51.7)	0.366
**BMI (kg/m^2), mean (SD)**	28.8 (4.7)	23.3 (1.2)	27.3 (1.3)	33.6 (3.9)	<0.001
**Cardiovascular risk factors, n (%)**					
Hypertension,	147 (65.6)	26 (57.7)	63 (67.0)	58 (68.2)	0.227
Time since diagnosis (years), mean (SD)	11.3 (10.4)	9.7 (9.4)	11.5 (10.2)	11.5 (10.9)	0.785
Hypercholesterolemia	33 (14.7)	8 (17.7)	12 (12.7)	13 (15.2)	0.398
Hypertriglyceridemia	170 (75.8)	32 (71.1)	72 (76.5)	66 (77.6)	0.146
Diabetes Mellitus	96 (42.8)	20 (44.4)	37 (39.3)	39 (45.8)	0.088
Time since diagnosis (years), mean (SD)	12.0 (8.5)	13.6 (10.0)	10.0 (6.6)	13 (9.2)	0.248

Characteristic		All (N=224)	Normal weight (n=45)	Overweight (n=94)	Obese (n=85)	P-value
Smoker		123 (54.9)	21 (46.6)	55 (58.5)	46 (54.1)	0.117
**Blood pressure (mmHg), mean (SD)**						
	SBP	136.5 (18.0)	133.6 (15.4)	136.9 (17.9)	137.6 (19.5)	0.470
	DBP	75.1 (11.0)	73.3 (12.1)	74.3 (10.3)	76.8 (11.0	0.161
**Lipid profile (mg/dL), mean (SD)**						
	TC	222.8 (44.2)	216 (37.5)	225.7 (46.1)	223.2 (45.3)	0.476
	TG	225.3 (101.8)	223.5 (100.5)	218.7 (90.5)	233.4 (114.2)	0.627
	LDL-c	127.4 (33.8)	119.7 (28.7)	131.6 (35.4)	126.8 (34.2)	0.146
	HDL-c	39.9 (10.1)	40.5 (10.3)	39.0 (9.5)	40.61 (10.5)	0.518

BMI: body mass index; DBP: diastolic blood pressure; HDL-c: high-density lipoprotein cholesterol; LDL-c; low-density lipoprotein cholesterol; SBP: systolic blood pressure; SD: standard deviation; TC: total cholesterol; TG: triglycerides
