# Supplementary material for: The cardiovascular polypill as baseline treatment improves lipid profile and blood pressure regardless of body mass index in patients with cardiovascular disease. The Bacus study
Source: PLoS One. 2023 Aug 25;18(8):e0290544. doi: 10.1371/journal.pone.0290544 (PMC10456133; doi:10.1371/journal.pone.0290544)
Supplement: Attachment — Submitted filename: BACUS- Rebuttal letter PLOS ONE June 2023.docx [file pone.0290544.s009.docx]

Thank you for inviting us to submit a revised draft of our manuscript entitled "**The Cardiovascular Polypill as Baseline Treatment Improves Lipid Profile and Blood Pressure Regardless of Body Mass Index in Patients with Cardiovascular Disease. The BACUS Study**" to PLOS ONE. We appreciate the time and effort that the reviewers have dedicated to providing insightful feedback on ways to strengthen our paper further. The manuscript has been modified to incorporate changes that reflect the detailed suggestions. In the revised version of the manuscript, modifications in response to the reviewer’s comments appear in track changes. A point-by-point description of how each query has been addressed is included in the following pages.

**Reviewer #1**

**There are several concerns which should be addressed.**

**1. This study is a single-center retrospective one. The authors should describe about patient selection in more detail. Were consecutive cases enrolled?**

As suggested by the reviewer, we have complemented the Methods section of the manuscript with additional information on data sources, patient selection, and data collection. The following changes were made:

**“Study design and data source**”; page 4, lines 96-100:

“This was an observational, single-center, retrospective, physician-led medical record review study conducted in the General Hospital of México (Mexico City, Mexico) Coronary Unit. The medical cardiologists responsible for patients requiring secondary CVD prevention treatment because of established coronary heart disease (CHD) retrospectively reviewed the medical records of consecutive eligible patients receiving the CV polypill between January 2013 and January 2019.”

**“Study population”**; page 5, lines 105-109:

All adult patients of both sexes attending the Coronary Unit from January 2013 to January 2019 had their medical records queried via the hospital’s electronic health record system. Of the eligible medical records, we identified in chronological order those from patients with a diagnosis of established CHD (i.e., unstable CHD or acute coronary syndrome [myocardial infarction and unstable angina]) requiring secondary CVD prevention treatment (…).

**“Data collection and study measures”** page 5, lines 121-126:

The data collected were part of the general cardiological assessments and management of the patients per routine clinical practice and national/local protocols and guidelines. Baseline characteristics, exposure, and outcomes were defined a priori to reduce the bias inherent to retrospective chart review studies. The qualitative and quantitative data were integrated into an Excel spreadsheet and deidentified from the electronic chart by removing the name, date of birth, and day of visit.

**2. In this study, patients with established coronary heart disease were enrolled. Please clearly define ‘established coronary heart disease’ in Methods section.**

We apologise for the lack of clarity regarding the diagnoses. In the new version of the manuscript that we are submitting, we have specified in the Study population subsection (see our response to the previous question) that coronary heart disease (CHD) includes patients with unstable CHD or acute coronary syndrome (myocardial infarction and unstable angina).

**3. Are there any data about cardiovascular events or death during follow-up periods? The information would be useful for understanding the clinical efficacy of polypill on secondary prevention.**

The reviewer is correct that data on recurrent cardiovascular events or cardiovascular death would provide valuable information on the efficacy of the CV polypill. Unfortunately, although this information was for sure recorded in the patient’s electronic medical charts, it was not extracted by the reviewers because these variables were not among the prespecified outcomes of the study. However, in the discussion section of the manuscript (page 17; lines 407-415), we mentioned the recent NEPTUNO study and the SECURE trial, where the polypill strategy reduced the relative risk of recurrent major adverse CV events (MACE: MI, stroke, revascularisation, and CV death) by 24% and the CV mortality by 33%.

References:

Gonzalez-Juanatey JR, et al. The CNIC-Polypill reduces recurrent major cardiovascular events in real-life secondary prevention patients in Spain: The NEPTUNO study. Int J Cardiol. 2022;361:116-23.

Castellano JM, et al. Polypill Strategy in Secondary Cardiovascular Prevention. N Engl J Med. 2022;387(11):967-77.

**4. Is there a difference in lipid profile and blood pressure between presence and absence of prior prescription of statin or RAS BI to CV polypill?**

We are providing two additional graphs to illustrate the answer to the reviewer’s question. In the upper panel A, you can see that the change in LDL-c levels among patients that were not treated with statins prior to switching to the CV polypill strategy was comparable to the change observed among those on equipotent and more potent statins. Please note that the apparent increase in LDL-c levels in patients with less potent statins is not evaluable because of the low sample size (n=17). In the lower panel B, you can see that the change in the systolic blood pressure (SBP) levels among patients that were not treated with antihypertensive drugs prior to switching to the CV polypill strategy was comparable to the change observed among those one or two antihypertensives. Please note that the apparent increase in SBP levels in patients with three antihypertensives is not evaluable because of the low sample size (n=4).

**5. In Figure 1 and Figure 2, Y-axis scale should start from zero.**

As recommended by the reviewer, Figures 1 and 2 have been scaled to include zero in the Y axes.

**6. The authors should explain S2 Fig in Results section.**

The information given in S2 Fig is described in the Results section of the manuscript on pages 9 to 10 (lines 199-207), with the following text:

“In normal weight patients, the previously prescribed statins were mostly equipotent to the 40 mg simvastatin of the CV polypill (58.1% of cases), while in overweight and obese subjects almost half of them were on more potent statins (50.6% and 53.7%, respectively) (**S2 Fig**). In contrast, the use of less potent prior statins was uncommon in all groups (16%, 4.4%, and 6.1% of cases of normal weight, overweight, and obese patients, respectively). Regarding RAAS inhibitors, agents less potent than the 5 mg or 10 mg ramipril contained in the CV polypill were more frequent than equipotent drugs only in normal weight patients (69.2% vs. 30.8%), whereas the prior use of equipotent drugs was more frequent than less potent drugs in overweight and obese patients (58.3% vs. 33.3% and 45.5% vs 36.4%, respectively; **S2 Fig**).”

**7. Detailed explanation about vascular age would be helpful for understanding its significance.**

As the reviewer suggested, we added information on how vascular age was estimated and its value in primary care settings. In the new version of the manuscript, we have added the following details:

Methods section, “Data collection and study measures” subsection; page 5, last paragraph (lines 132-134):

“With the obtained data, we calculated the vascular age, defined as the CVD risk of an individual -derived from the Framingham risk tables and SCORE scale equations- transformed to the age of a person with the same risk but controlled risk factors [28].”

Discussion section, page 17, lines 381-387:

“Vascular or heart age is an emerging health metric in primary care defined as the CVD risk of an individual transformed to a person’s age if the same risk factors were optimally controlled [42]. This allows translating the abstract concept of 10-year CV risk into quite an understandable message for the patient that leads to more significant decreases in CV risk factors than the standard of care [43]. For instance, it would inform a 40-year-old male smoker with hypertension and hypercholesterolemia (moderate risk as per SCORE scale) that he has a vascular age comparable to a healthy 63-year-old man [43].”

References:

28. Cuende JI, et al. How to calculate vascular age with the SCORE project scales: a new method of cardiovascular risk evaluation. Eur Heart J. 2010;31(19):2351-8. doi: 10.1093/eurheartj/ehq205. PMID: 20584778

42. D’Agostino, et al. General Cardiovascular Risk Profile for Use in Primary Care. Circulation. 2008;117(6):743-53.

43. Cuende JI. Vascular Age Versus Cardiovascular Risk: Clarifying Concepts. Rev Esp Cardiol (Engl Ed). 2016;69(3):243-6.

**8. The mean time treatment with the CV polypill and follow-up period should be shown in each subgroup in Table 1.**

As recommended by the reviewer, we have added the mean time on treatment with the CV polypill in Table 1 (page 9):

|  | All  (N=479) | Normal weight  (n=80) | Overweight  (n=216) | Obese  (n=183) | P-value |
| --- | --- | --- | --- | --- | --- |
| Time on treatment (months), mean (SD) | 56.64 (18.7) | 54.21 (16.2) | 55.90 (15.6) | 58.60 (22.7) | 0.169 |

**9. What kind of statistical test was performed in this study?**

The statistical tests used in the study were described in the Statistical Analysis section of the manuscript. However, we understand they needed to be more precise, and we have reworded this section for clarity. The new paragraph reads as follows (Page 6; lines 148-160):

“(…) The effectiveness of the CV polypill was assessed through the difference between baseline and the last follow-up visit in the mean value of the lipid profile (TC, HDL-c, LDL-c, and TG), lipid ratios (i.e., TC/HDL-c and LDL-c/HDL-c), blood pressure (i.e., SBP and DBP), and vascular age; the statistical significance of the change was evaluated by a Student's t-test for repeated measures. Moreover, the change in the proportion of patients with on-target LDL-c levels (i.e., <70 mg/dL) and patients with normal or borderline high TG levels (<150 and <200 mg/dL, respectively) were compared to baseline between groups by a Chi-square test. Please note that the target levels for LDL-c and recommended levels for TG were those of the 2016 European Society of Cardiology (ESC) guidelines on lipid management [30], since this study was performed before the latest 2019 ESC guidelines were published [31]. Additionally, the difference in the proportions of patients who achieved the target BP (i.e., <140/90 mmHg or <130/85 mmHg for patients with T2DM) between baseline and after the use of the CV polypill and, among them, the percentage that needed concomitant medication or not was also assessed through the Chi-square test.(…)”

**Reviewer #2**

**As authors addressed the study comes with significant limitations.**

We understand that the study has several limitations, and the reviewer recommended the rejection of the manuscript. However, we would like to take this opportunity to respond to his concerns to improve the quality of the manuscript as much as possible.

For this, we have grouped the questions between the limitations of the study design and other issues raised by the reviewer.

**1. Limitations of the study identified by the reviewer.**

**1. The study is a retrospective design.**

**2. Data were extracted from hospital medical records, and methodological bias cannot be excluded.**

**3. There is no control group in the study.**

**4. Authors state that inaccuracies in data collection due to the design cannot be excluded.**

**5. Selection bias is likely for polypill that favoring patients for polypill will be the ones with poor compliance.**

We agree with the reviewer that the design of the study, namely a retrospective chart review with a convenience sample (selection of existing suitable cases), is subject to several limitations concerning the generalizability of results. The reviewer listed the issues we mentioned in the manuscript's discussion section.

While there remain many notable limitations to retrospective chart review research, it is a relatively inexpensive option to research readily accessible existing data, and it is a practical method particularly useful in cases where information is limited (small sample size).

Reference:

Vassar M, Holzmann M. The retrospective chart review: important methodological considerations. J Educ Eval Health Prof. 2013;10:12

**2. Other issues raised by the reviewer.**

**6. No study protocol is present due to the design.**

Indeed, no protocol was written, although the baseline characteristics, exposure and outcomes that the reviewers had to extract from the medical charts were predefined.

**7. Patients were not treated adequately prior to the polypill.**

Yes, this was also mentioned as a study limitation that could have resulted in overestimating the CV polypill effect on the studied outcomes.

**8. The study has limited novelty. Several polypills have been studied in numerous research trials in comparison to placebo or usual care.**

This is indeed true. However, our study was not designed to compare the CV polypill approach to other strategies (such as usual care or lose components) to identify predictors of response. All patients were treated with the CV polypill, and the outcomes were compared between patients with different BMI groups to evaluate whether BMI could influence the clinical response. As raised by reviewer #3 (question 2), this is the first study assessing the impact of the CV polypill as a secondary prevention strategy as a function of weight stratification.

**9. Previous clinical trials have measured adherence rates, adverse events and risk factor control.**

We are aware that several trials have evaluated these outcomes. These studies are mentioned in the discussion section for comparison with our findings regarding risk factor control and give a complete view of reported outcomes not included in our study (e.g., adherence rates or recurrence of CV events).

**10. This trial design would not have the power to test the impact of polypill on clinical outcomes.**

The small sample size was not mentioned as a limitation of the study. In the new version of the manuscript that we are submitting, we have added this in the discussion section (page 17, lines 408-410):

“One weakness of the study is that the sample size was relatively small, thus with low statistical power potentially resulting in a type II error limiting the generalizability of the results to larger populations of patients. (…)”

**11. Use of polypill will come with concerns i.e. that a side-effect of any one of the components can lead to discontinuation of all drugs.**

The reviewer is right that this is a common concern among physicians. However, the randomized controlled FOCUS study reported a similar proportion of adverse events in post-myocardial infarction patients treated with the CV polypill or with separate components, with treatment discontinued by 4% of patients in each arm (Castellano et al., 2014). A similar rate of adverse events was also reported in the randomized controlled SECURE trial, comparing the polypill-based strategy with usual care in patients in secondary prevention (Castellano et al., 2022).

References:

Castellano JM, et al. A polypill strategy to improve adherence: results from the FOCUS project. J Am Coll Cardiol. 2014;64(20):2071-82.

2. Castellano JM, et al. Polypill Strategy in Secondary Cardiovascular Prevention. N Engl J Med. 2022;387(11):967-77.

**12. Clinicians by training are taught to titrate dosages to ‘treat to target’ various risk factors. The key worry for most practitioners has been the inconvenience of dose adjustments with a polypill. The lack of ability to fine tune one component of the polypill if the therapeutic goals are not reached is a constant concern.**

Indeed, the inability to titrate dosage when the desired therapeutic effect is not achieved is a documented driver of the physician’s defiance of fixed-dose combination prescription (Roy et al., 2017). However, the CV polypill must be understood as a baseline treatment and other BP-lowering or lipid-lowering drugs can be added whenever needed in patients with suboptimal control (Coca et al., 2020; Grigorian-Shamagian et al., 2021).

References:

Roy A, et al. Strengths and Limitations of Using the Polypill in Cardiovascular Prevention. Curr Cardiol Rep. 2017;19(5):45

Coca A, et al. A practical approach to switch from a multiple pill therapeutic strategy to a polypill-based strategy for cardiovascular prevention in patients with hypertension. J Hypertens. 2020;38(10):1890-98.

Grigorian-Shamagian L, et al. Practical Decision Algorithms for the Use of the Cardiovascular Polypill in Secondary Prevention in Europe. Front Cardiovasc Med. 2021;8:663361.

**13. Drug interactions are of concern. I.e. conflicting reports exist on the concomitant use of aspirin and ACE inhibitors**

The reviewer is right that the safety of this association has been questioned because aspirin may counteract the beneficial effect of angiotensin-converting enzyme (ACE) inhibitors. However, as the reviewer highlights, most evidence comes from observational studies, and the results are conflicting. Nevertheless, the recommendation is to limit the aspirin dosage in long-term therapy to ≤100 mg/day doses (the dose that the CV polypill contains), which may mitigate the adverse interaction (Barbash et al., 2000; Zanchetti et al., 2002).

References:

Barbash IM, et al. Possible Interaction Between Aspirin and ACE Inhibitors: Update on Unresolved Controversy. Congest Heart Fail. 2000;6(6):313-18

Zanchetti A, et al. Low-dose aspirin does not interfere with the blood pressure-lowering effects of antihypertensive therapy. J Hypertens. 2002;20(5):1015-22.

**Reviewer #3**

**In my discussion I have asked for clarifications and suggested a focused discussion that highlights your findings. I have also suggested that your findings be compared with degree of BP and lipid-lowering in light of previous studies as outlined in my review. All these are minor adjustments.**

We thank the reviewer for the thorough review of our manuscript and the valuable comments raised.

**1. Weakness in the study design:**

**1.1 Absence of Control Group. Lack of control group for comparison: Patient with multi-pill regimen in each weight category and their outcomes.**

The reviewer is right that the lack of a comparator (within-subjects design) is subject to several limitations concerning the generalizability of results. Our study was not designed to compare the CV polypill approach to other strategies (such as usual care) to identify predictors of response. Conversely, this was a case series (uncontrolled study with a convenience sample) design, thus, all patients were treated with the CV polypill, and the outcomes were compared between patients with different BMI groups to evaluate whether BMI could influence the clinical response.

The lack of a control group was acknowledged as a limitation of the study in the discussion section of the manuscript. Still, we reworded the sentence to clarify that this design is subject to selection bias. The new sentence reads as follows (page 18; lines 427-431):

“Moreover, the study had a non-randomized design without a control group (case series) prone to selection bias. Indeed, the decision to initiate or switch to the CV polypill strategy was made by the treating physician (and the patient) while providing usual care. As such, the results of the study may be susceptible to selection bias favoring patients for whom the CV polypill was best suited (e.g., poorly adherent) according to the preferences or experience of individual doctors or local protocols. (…)”

**1.2. Exclusion criteria not outlined: What were the total number of records viewed, how many were excluded on the basis of lack of visit history and lack of labs or incomplete records. What measures were put in to prevent selection bias.**

As suggested by the reviewer, in the new version of the manuscript, we briefly summarize manuscript the total number of records viewed and how many were excluded in the results section (page 7, lines 165-167):

“Out of 595 eligible patients who were previously treated with the standard of care (i.e., multi-pill strategy), 479 with an established CHD who switched to or initiated treatment with the CV polypill and had complete records were included in the study.”

Regarding the measures to prevent selection bias, this has been included in the manuscript as per reviewer #1 suggestion. Please see our response to the next question for details.

**1.3. Method of data collection not outlined.**

As noted by reviewer #1 (question 1), we have added additional information on the data collection:

**“Data collection and study measures”** page 5, lines 121-126:

The data collected were part of the general cardiological assessments and management of the patients per routine clinical practice and national/local protocols and guidelines. To reduce the bias inherent to retrospective chart review studies, baseline characteristics, exposure, and outcomes were defined a priori. The qualitative and quantitative data were integrated into an Excel spreadsheet and deidentified from the electronic chart by removing the name, date of birth, and day of visit.”

**1.4. Adherence to treatment data is not included: The data appears to be homogeneous and it appears that all the patients, in all the groups were taking polypill religiously. Adherence to treatment has been a major factor impacting secondary prevention strategies in low to middle-income countries. In multipill world adherence to medication is as low as 40% and as high as 80%. In the SECURE polypill Trial, several barriers to adherence were outlined including complexity of regimen, poly-pharmacy, coexisting illness, Age of the patient, socioeconomic (affordability and availability of medications), insurance coverage, and educational. In the SECURE Trial adherence rate for polypill was 74% as compared to multiple pills 63% at 24 months (1). In the FOCUS Project, with a much stringent criteria of adherence was used, the adherence rate for polypill was 65% at 9 months (2).**

Medication adherence has indeed been pointed out as one of the reasons why the CV polypill approach is a good strategy for improving lipids and blood pressure control (Baumgartner et al., 2020; Rao et al., 2022; Memon, 2023). The reviewer is correct that the manuscript does not give data on medication adherence. This is because adherence was not quantified through a structured questionnaire (e.g., the Morisky Medication Adherence Scale) or pill count but self-reported by the patient when asked by the treating physician and usually documented as unstructured free text in the medical records. Therefore, we think it would have been biased and unrealistic to give data on adherence rates based on the reviewed medical charts. As we understand that the lack of adherence data might undermine the interpretation of the results, it has been added as a further limitation of the study (page 18, lines 411-423):

“Firstly, data were extracted from hospital medical records, and it is possible that not all pertinent risk factors or predictor variables were identified or subsequently recorded. For instance, several meta-analyses have shown that the CV polypill strategy has a significant positive impact on medication adherence, with more than 76% of patients showing medication adherence [46-48]. This was also documented in trials using the same CV polypill as in our study in patients in secondary prevention. For instance, in the randomized FOCUS trial [49], the adherence rate for the CV polypill was 65.7% at 9 months vs 55.7% in patients taking the three drugs separately. In the recent SECURE trial [24], 74.1% of the patients in the polypill group and 63.2% of those in the usual-care group were adherent after 24 months of follow-up. In the present research, medication adherence was not included in the analyses because it was self-reported by the patient when asked by the treating physician and documented as unstructured free text in the medical records. However, adherence rates were not expected to differ between weight groups because all patients were treated with the CV polypill.(…)”

References:

24. Castellano JM, et al. Polypill Strategy in Secondary Cardiovascular Prevention. N Engl J Med. 2022;387(11):967-77.

46. Baumgartner A, et al. Does the Polypill Improve Patient Adherence Compared to Its Individual Formulations? A Systematic Review. Pharmaceutics. 2020;12.

47. Memon RA, et al. Effect of the Polypill on Adherence and Prevention of Cardiovascular Diseases in Patients With or at High Risk of Cardiovascular Diseases: A Meta-Analysis of Randomized Controlled Trials. Cureus. 2023;15(1):e34134.

48. Rao S, Jamal Siddiqi T, Khan MS, Michos ED, Navar AM, Wang TJ, et al. Association of polypill therapy with cardiovascular outcomes, mortality, and adherence: A systematic review and meta-analysis of randomized controlled trials. Prog Cardiovasc Dis. 2022;73:48-55. doi: 10.1016/j.pcad.2022.01.005. PMID: 35114251

49. Castellano JM, Sanz G, Penalvo JL, Bansilal S, Fernandez-Ortiz A, Alvarez L, et al. A polypill strategy to improve adherence: results from the FOCUS project. J Am Coll Cardiol. 2014;64(20):2071-82.

**2. How does this data add to the body of literature in the field of polypill and secondary prevention strategy? Most studies in polypill arena have been prospective randomized trials comparing polypill with placebo, as primary prevention strategy. In these trials a reduction in BP in these studies ranged between 5-10 mmHg and LDL reduction of 30-50 mg/dl (3). In the more recent Trials Polypill has been used as a secondary prevention strategy compared to usual multiple pill strategy. BP reduction was 8-12 mm Hg and LDLc reduction 10.3% (4). The reduction and BP and LDLc are in this study are consistent with prior studies. Previous studies have not looked impact of pollypill as a secondary prevention strategy as a function of weight stratification. This adds to the body of knowledge.**

We thank the reviewer for providing insight into the contribution of the study to the existing literature. In the introduction, we have stressed the limited available data on the use of the CV polypill in secondary prevention. To update the manuscript with comparable studies using the CV polypill in secondary prevention, we have added the appropriate references. Moreover, in the discussion section, we have added comparable data reported in other studies, and in the conclusions, we have added weight stratification as a novelty in the field of CV polypills. The changes made are described in detail below:

Introduction, page 4, lines 82-86:

“(…) However, most of the available studies implementing CV polypills were prospective randomized trials (RCTs) using placebo as a comparator in patients in primary prevention, with limited data on its impact on clinical outcomes in the secondary prevention setting [21-23]. Moreover, secondary prevention data arose from studies mainly conducted in European populations [24, 25].”

Discussion section, page 16, lines 340-351:

“(…) After treatment with the CV polypill, the mean levels of lipid parameters (TC, TG, and LDL-c) decreased 31% to 43% across all BMI groups, the mean HDL-c levels increased between 13% and 14%, and the lipid ratios, indicative of atherogenic dyslipidemia, almost halved (i.e., TC/HD-c and LDL-c/HDL-c decreased between –39% and –50% across groups). This is in line with the 25-30% reductions in TC, TG, and LDL-c reported by a prospective, hospital-based study conducted in the Mexican population, assessing the effectiveness of the CV polypill after 6 months in patients with myocardial infarction [35, 36]. Our findings also agree with the SORS observational and prospective registry study conducted in Mexico, including patients in secondary prevention (22-23% reduction in TC and LDL after 12 months) [37]. Finally, the LDL-c reduction was higher in our study than in the retrospective Spanish NEPTUNO study (15.4% reduction after 2 years) [25] and higher than the European SECURE randomized trial (10.3% reduction after 3 years) [24], both evaluating patients with established CVD.(…)”

Discussion section, page 16, lines 368-391:

“Regarding BP control, we observed a 10–11% decrease in SBP and 5–6% in DBP after polypill treatment, with no differences between weight groups, such that more than 80% of patients were at recommended levels at the end of the study. These results align with those reported by the SORS hospital-based observational, prospective Mexican study, including patients in secondary prevention (11.5% and 10% reduction in SBP and DBP, respectively) [37]. They also align with the reductions reported in the retrospective NEPTUNO study conducted in patients with established CVD (10.1% and 5.9% reduction in SBP and DBP, respectively) [25].

References:

21. Sharif M, et al. One pill for them all: polypill therapy for the prevention of cardiovascular disease. Expert analysis. American College of Cardiology. Mar 2019, 2021. Available at https://www.acc.org/latest-in-cardiology/articles/2021/03/19/11/59/one-pill-for-them-all Accessed 19 May 2023.

22. Sadeghi M, et al. Fixed Dose Combination Therapy for Secondary Prevention of Major Cardiovascular Events: Review of Literature and Design for Persian Polypill, A Randomized Clinical Trial. Curr Probl Cardiol. 2022;47(6):100872.

23. Di Fusco SA, et al. The polypill strategy in cardiovascular disease prevention: It's time for its implementation. Prog Cardiovasc Dis. 2023.

24. Castellano JM, et al. Polypill Strategy in Secondary Cardiovascular Prevention. N Engl J Med. 2022;387(11):967-77.

25. Gonzalez-Juanatey JR, et al. The CNIC-Polypill reduces recurrent major cardiovascular events in real-life secondary prevention patients in Spain: The NEPTUNO study. Int J Cardiol. 2022;361:116-23.

35. Mendez-Garcia LA, et al. Six Month Polypill Therapy Improves Lipid Profile in Patients with Previous Acute Myocardial Infarction: The Heart-Mex Study. Arch Med Res. 2019;50(4):197-206.

36. Gomez-Alvarez E, et al. The CNIC-polypill improves atherogenic dyslipidemia markers in patients at high risk or with cardiovascular disease: Results from a real-world setting in Mexico. Int J Cardiol Heart Vasc. 2020;29:100545.

37. Castellano JM, et al. Clinical Effectiveness of the Cardiovascular Polypill in a Real-Life Setting in Patients with Cardiovascular Risk: The SORS Study. Arch Med Res. 2019;50(1):31-40.

**3. Overreach: The conclusion states that irrespective of BMI and age, polypill as a baseline therapy as a secondary prevention strategy in reducing coronary artery risk factors is beneficial. (line 418). However, the authors have not compared different age groups in there results.**

In our study, we included a subanalysis in patients older than 65 years, but the reviewer is right that we did not compare the results with other age groups. This statement in the conclusion section has been rewritten, and the sentence now does not include the word age (Conclusions section; page 19; lines 446-449):

“In the Mexican population, where hypertension and hypercholesterolemia are prevalent and the treatment rates are low, the current findings add to a growing body of literature on the use of the CV polypill as baseline therapy for secondary prevention, providing evidence that it is a beneficial strategy regardless of the patient’s BMI.”

**4. Missing literature: Authors have missed a number of studies in the use of polypill as a primary prevention strategy and % reduction LDL and mmHG reduction BP in those studies for comparison to validate their findings. A summary was published by Sharif etal. Expert Analysis on Polypill. 2021 JACC. March 19 issue**

We thank the reviewer for the valuable comments on the missing literature. As described in the previous responses, we have added data from available studies in secondary prevention and references to different reviews regarding the use of CV polypills in the primary and secondary prevention setting.

**5. Discussion Section:**

**5.1. Lines 348 to 356 are moving the discussion away from the topic and the authors should consider removing them.**

As suggested by the reviewer, this paragraph has been deleted from the manuscript.

**5.2. Section (lines 357 to 376) should be simplified as it detracts the reader from the point being made.**

As suggested by the reviewer, this paragraph has been simplified and now reads as follows (lines 374-380):

“Moreover, 55% of patients at target BP used concomitant anti-hypertensive medication to remain controlled in addition to the baseline CV polypill. This also aligns with another study reporting that 62% of patients still need additional BP treatment on top of the CV polypill [36]. Nevertheless, we observed that the proportion of subjects with BP on target increased compared to prior treatment in both groups, patients needing and not needing concomitant medication. This result demonstrates that regardless of the prior antihypertensive treatment, the CV polypill increased the overall proportion of controlled patients.”

**Reviewer #4**

**a) May I suggest using the word 'control' in the limitation section. As you rightly pointed out, you cannot prove cause-effect here as the study was a retrospective study design, with undoubtedly no control population.**

In the limitations section of the manuscript, the lack of a control group is now mentioned as a weakness of the study in two different instances (page 18, lines 427-428 and 433-435):

“Moreover, the study had a non-randomized design without a control group (case series) prone to selection bias.(…)”

“Additionally, because of the retrospective design and lack of a control group, we can only establish an association and not a cause-effect between the CV polypill strategy and the observed improvement in risk factors. (…)”

**b) Would also go into costings here, as this may also be an issue in widespread implementation of such a therapy.**

As recommended by the reviewer, we have added a paragraph in the discussion section to describe the results of economic analyses conducted with CV polypills (lines 399-407):

“The economic costs of implementing the CV polypill strategy were out of the scope of this study, but the price of the polypill is generally perceived as a barrier in low/medium socioeconomic populations with limited financial resources of the health system [45]. However, a recent meta-analysis of socioeconomic studies concluded that polypills resulted in improved adherence and quality of life at a price equal to or lower than multiple monotherapies, with the price (which was below the commonly accepted thresholds or cost saving in both primary and secondary prevention) as one of the key drivers of the cost-effectiveness [16]. Similarly, a cost-utility analysis conducted in Portugal documented that the CV polypill was consistently cost-effective compared with monocomponents in secondary prevention [17].”

References:

16. Jahangiri R, et al. Cost-effectiveness of fixed-dose combination pill (Polypill) in primary and secondary prevention of cardiovascular disease: A systematic literature review. PLoS One. 2022;17(7):e0271908.

17. Aguiar C, et al. Cost-Effectiveness of the CNIC-Polypill Strategy Compared With Separate Monocomponents in Secondary Prevention of Cardiovascular and Cerebrovascular Disease in Portugal: The MERCURY Study. J Health Econ Outcomes Res. 2022;9(2):134-46.

45. Yusuf S, et al. Use of secondary prevention drugs for cardiovascular disease in the community in high-income, middle-income, and low-income countries (the PURE Study): a prospective epidemiological survey. Lancet. 2011;378(9798):1231-43.

**3) Can you also go into hospitalisation/revascularisation/deaths? Is the proportion of participants who succumbed to these outcomes similar to statistics in the preceding years? Any differences compared to the ones who discontinued the polypill?**

A similar question was raised by reviewer #1 (question 3). The reviewer is right that data on hospitalisation, revascularisation procedures, and CV deaths would provide valuable information on the efficacy of the CV polypill. Unfortunately, although this information was for sure recorded in the patient’s electronic medical charts, it was not extracted by the reviewers because these variables were not among the prespecified outcomes of the study. However, in the discussion section of the manuscript (page 17; lines 390-398), we mentioned the recent NEPTUNO study and the SECURE trial, where the polypill strategy reduced the relative risk of recurrent major adverse CV events (MACE: MI, stroke, revascularization, and CV death) by 24% and the CV mortality by 33%.

References:

Gonzalez-Juanatey JR, et al. The CNIC-Polypill reduces recurrent major cardiovascular events in real-life secondary prevention patients in Spain: The NEPTUNO study. Int J Cardiol. 2022;361:116-23.

Castellano JM, et al. Polypill Strategy in Secondary Cardiovascular Prevention. N Engl J Med. 2022;387(11):967-77.
